# Inflammatory activation and endothelial dysfunction markers in patients with permanent atrial fibrillation: a cross-sectional study

**DOI:** 10.18632/aging.103149

**Published:** 2020-05-03

**Authors:** Carlo Domenico Maida, Sonya Vasto, Domenico Di Raimondo, Alessandra Casuccio, Valerio Vassallo, Mario Daidone, Alessandro Del Cuore, Gaetano Pacinella, Anna Cirrincione, Irene Simonetta, Vittoriano Della Corte, Salvatore Rizzica, Giulio Geraci, Antonino Tuttolomondo, Antonio Pinto

**Affiliations:** 1 U.O.C di Medicina Interna con Stroke Care, Dipartimento di Promozione della Salute, Materno-Infantile, di Medicina Interna e Specialistica di Eccellenza “G. D’Alessandro”, University of Palermo, Palermo, Italy; 2 Dipartimento di Scienze e Tecnologie Biologiche Chimiche e Farmaceutiche, University of Palermo, Palermo, Italy; 3 Dipartimento di Promozione della Salute, Materno-Infantile, di Medicina Interna e Specialistica di Eccellenza “G. D’Alessandro”Section of Public Health Epidemiology and Preventive Medicine, University of Palermo, Palermo, Italy; 4 Molecular and Clinical Medicine PhD Programme, University of Palermo, Palermo, Italy; 5 Unit of Nephrology and Hypertension, European Society of Hypertension Excellence Center, Dipartimento di Promozione della Salute, Materno-Infantile, di Medicina Interna e Specialistica di Eccellenza “G. D’Alessandro”, University of Palermo, Palermo, Italy

**Keywords:** atrial fibrillation, cytokines, inflammation, endothelial dysfunction, structural heart disease

## Abstract

In recent years a growing body of evidence supported the role of inflammation in the initiation, maintenance and outcome of atrial fibrillation (AF). Nevertheless, despite a large amount of information, whether AF or the underlying structural heart disease (SHD) is the cause of the inflammatory process is still under debate. We, therefore, sought to determine if the inflammatory process reflect an underlying disease or the arrhythmia ‘per se’. We evaluated plasma levels of soluble Interleukin 2 Receptor Alpha (sIL-2Rα), TNF-α and IL-18 in 100 consecutive patients with permanent AF, (43 with a SHD and 57 without a SHD) compared to 121 age and sex-matched controls which had normal sinus rhythm. We also evaluated the endothelial function in both groups of patients using reactive hyperemia index (RHI) values measured by Endo-PAT2000. Compared to controls, AF patients showed higher circulating levels of inflammatory markers and a lower mean value of RHI. At multiple logistic regression analysis, the inflammatory markers and RHI were significantly associated with AF presence, whereas ROC curve analysis had good sensitivity and specificity in inflammatory variables and RHI for AF presence. No significant association was observed in the group of permanent AF patients, between inflammatory markers and the presence of an underlying SHD. These findings could help to clarify the role of inflammation in subjects with AF and suggest that the markers of systemic inflammation are not associated with the underlying cardiovascular disease, rather with the atrial fibrillation ‘per se’.

## INTRODUCTION

Atrial fibrillation (AF) is the most common cardiac rhythm disorder and it represents one of the major causes of heart failure, stroke, sudden death and cardiovascular morbidity worldwide [1]. In recent years, several studies [2–4] have described a strong association between AF and inflammation, suggesting the key role of the inflammatory process in the initiation, maintenance and outcome of the arrhythmia itself. Multiple inflammatory markers and mediators have been found to be elevated in patients with AF, including C-reactive protein (CRP), interleukin (IL)-2, IL-6, IL-8, monocyte chemoattractant protein (MCP)-1, tumor necrosis factor (TNF)-α. Furthermore, inflammatory infiltrates compatible with a diagnosis of myocarditis (lymphomononuclear infiltrates) has been demonstrated by several studies [5, 6] performing atrial and ventricular endomyocardial biopsies in patients with "lone AF" (LAF), suggesting the histological inflammatory substrate of this arrhythmia.

Nevertheless, despite a large amount of information, whether the cause of the inflammation is the arrhythmia itself or the underlying structural heart disease (SHD) is still under debate. Only a few studies [7, 8] investigated this field assessing the circulating levels of CRP mostly in subjects with LAF and with SHD, without clarifying if this marker is associated with the AF ‘per se’ or rather with the underlying cardiovascular disease. Besides, these studies have investigated the inflammatory pathway of atrial fibrillation patients only by determine CRP plasma levels, and in recent years numerous evidence [9] suggest to avoid the use of the historical term “lone AF”.

Considering the active link between the arrhythmia and the inflammatory process, reported by previous studies [2–6], we hypothesized that the inflammatory activation of AF subjects may represent the mirror of the arrhythmia ‘per se’, regardless of the presence of an underlying abnormality of heart structure. We, therefore, aimed to investigate patients with permanent AF and compared their "inflammatory cytokines milieu" to that of controls in sinus rhythm evaluating the association between inflammatory markers and underlying SHD.

Furthermore, in recent years AF has been associated with endothelial dysfunction [10, 11]. Besides, accumulating evidence supports the role of inflammatory cytokines in endothelial dysfunction [12]. Thus, the purpose of the study was also to assess the endothelial function in our AF cohort compared to controls in sinus rhythm, through the use of reactive hyperemia peripheral arterial tonometry (RH-PAT), and to evaluate the relationship between inflammatory variables and this vascular health marker.

## RESULTS

**Figure 1:**
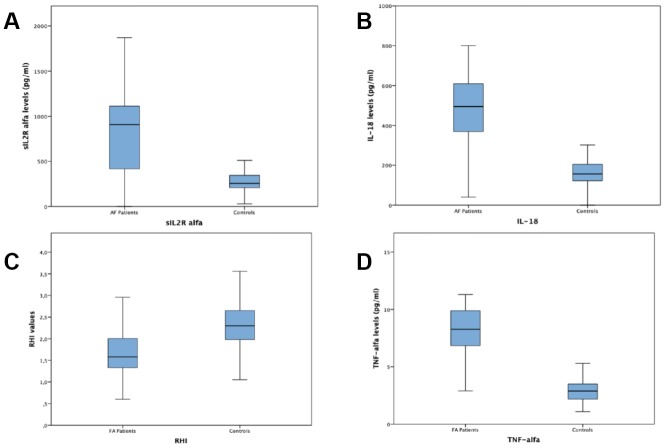
**Plasma values of sIL-2Rα, IL-18, TNF-α and RHI values in atrial fibrillation subjects vs. controls.** (**A**) Plasma values of sIL-2Rα in atrial fibrillation subjects compared with controls. (**B**) Plasma values of IL-18 in atrial fibrillation subjects compared with controls. (**C**) RHIvalues in atrial fibrillation subjects compared with controls. (**D**) Plasma values of TNF-α in atrial fibrillation subjects compared with controls.

A total of 100 subjects with permanent AF and 121 control subjects in sinus rhythm without any history of AF, were enrolled. General and laboratory characteristics of patients with AF and of subjects in sinus rhythm and without any history of AF are listed in Table 1. Among AF patients 47 (%) had a SHD while 53 (%) had no SHD. Characteristics and differences of AF patients with and without SHD are listed in Table 2. Subjects with AF showed in comparison to subjects without AF a significantly higher frequency of hypertension (84 vs 58.08%; p <0.001) and diabetes mellitus (34% vs 30.25%; p <0.019), higher mean cholesterol blood levels (148.94 ± 40.13 mmol / L vs 133.74 ± 35.17 mmol / L; p <0.003), higher mean HbA1c blood levels (6.16 ± 1.15 vs 5.31± 1.44; p <0.001), higher mean microalbuminuria values (95.56 ± 39.77 vs 61.94 ± 31.73; p <0.025) and higher mean left atrium diameter (45.99±9.05vs 38.47 ± 7.57; p<0.001). Subjects with AF had lower mean heart rate values (80.01 ± 16.64bpm vs 88.15 ± 12.39 bpm; p <0.001), lower mean SBP values (127.33 ± 15.57 mmHg vs 131.28 ± 10.45 mmHg; p <0.026), lower mean left ventricular ejection fraction values (50.39 ± 11,29% vs 55.58 ± 9.40%; p<0.001) and a lower mean value of RHI (1.76 ± 0.83 vs. 2.34 ± 0.68; p = 0.001) (Figure 1). Regarding of inflammatory markers, AF patients had higher plasma values of IL 18 (442.72±172.34 vs 172.85±109.69; p<001), TNF α (7.85±3.05 vs 2.99±0.97;p<0,001) and sIL-2Rα (867.22±391.40 vs 276.28±95.82; p< 0,001) compared with controls (see figure 1). Subjects with AF and SHD showed in comparison to AF subjects with NSHD a significantly higher mean age (77.5 ± 9.3 vs 70.4 ± 13.3; p= 0.003) and higher mean left atrium diameter (53.9±6.9vs 38.9 ± 2.5; p<0.005). Subjects with AF and SHD had lower mean left ventricular ejection fraction values (39.5 ± 4.4% vs 60 ± 4.9%; p<0.0005) and a lower mean value of RHI (1.5 ± 0.4 vs. 2 ± 1; p = 0.002). Regarding of inflammatory markers, AF patients with SHD had higher plasma values of TNF α (8.6±1.9 vs 7.2±3.7;p=0,021) and sIL-2Rα (964.9±334.6 vs 782.4±419.6; p= 0,020) compared with AF patients with NSHD.

**Table 1:** Demographic, clinical and laboratory variables in subjects with atrial fibrillation and in controls.

**Variables**	**AF Patients (n=100)**	**Controls (n=121)**	**p**
**Age (years) (mean± SD)**	73.70± 12.05	74.98±9.63	0.383
**M/F**	54/46	73/48	
**SBP (mmHg) (mean± SD)**	127.33 ± 15.57	131.28±10.45	0.026
**DBP (mmHg) (mean± SD)**	71.48±10.36	72.917±9.93	0.295
**Weight (Kg) (mean± SD)**	75.18±14.24	77.90±11.03	0.111
**BMI (kg/m2) (mean± SD)**	26.85±4.66	27.67±3.81	0.152
**Hypertension (n/%)**	84/84	48/39,66	0.001
**Diabetes (n/%)**	34/34	25/20.66	0.019
**Dyslipidaemia (n/%)**	35/35	41/33,88	0.093
**SHD (n/%)**	47/47	30/24,79	0.019
**Heart rate (bpm) (mean± SD)**	80.01±16.64	88.15±12.39	0.001
**Left atrium diameter (mm) (mean± SD)**	45.99±9.05	38.47±7.57	0.001
**LVEF (%) (mean± SD)**	50.39±11.29	55.58±9.40	0.001
**Total cholesterol (mMol/L) (mean± SD)**	148.94±40.13	133.74±35.17	0.003
**LDL cholesterol (mMol/L) (mean± SD)**	82.76±33.30	83.75±33.95	0.828
**Triglycerides (mmol/L) (mean± SD)**	90.52±40.67	84.46±28.57	0.196
**HBa1C (mean± SD)**	6.16±1.15	5.31±1.44	0.001
**Micro albuminuria (mean± SD)**	95.56±39.77	61.94±31.73	0.025
**eGFR (ml/min) (mean± SD)**	72.47± 17.09	70.70± 19.87	0.477
**ACE inhibitors or ARB (n/%)**	61/61	47/38.84	0.001
**Beta blockade (n/%)**	50/50	37/30.57	0.003
**Calcium channel blockers (n/%)**	26/26	33/27.27	0.477
**Antiarrhythmic drugs (n/%)**	27/27	9/7.43	0.001
**Antiplatels drugs (n/%)**	37/37	32/26.44	0.109
**Anticoagulant drugs (n/%)**	53/53	1/0.82	0.001
**Diuretic (n/%)**	49/49	14/11.57	0.001
**Statin (n/%)**	34/34	37/30.57	0.665
**RHI (mean± SD)**	1.76±0.83	2.34±0.68	0.001
**IL-18 pg/mL (mean± SD)**	442.72±172.34	172.85±109.69	0.001
**sIL2Ralfa pg/mL (mean± SD)**	867.22±391.40	276.28±95.82	0.001
**TNF-alfa pg/mL (mean± SD)**	7.85±3.05	2.99±0.97	0.001

**Table 2:** Demographic and clinical characteristics of atrial fibrillation patients with and without SHD.

**AF Patients (n=100)**	**SHD (n= 47)**	**NSHD (n= 53)**	**p**
**Age (years) (mean± SD)**	77.5 ± 9.3	70.4 ± 13.3	0.003
**M/F**	27/20	27/26	0.515
**Hypertension (n/%)**	40/85.1	44/83	0.776
**Diabetes (n/%)**	16/34	18/33,4	0.642
**Dyslipidaemia (n/%)**	18/38,3	17/32,1	0.515
**Weight (Kg) (mean± SD)**	76.2 ± 13.9	74.2 ± 14.7	0.489
**BMI (kg/m2) (mean± SD)**	27.4 ± 4.9	26.3 ± 4.4	0.248
**SBP (mmHg) (mean± SD)**	127.0 ± 16.5	127.6 ± 14.8	0.863
**DBP (mmHg) (mean± SD)**	70.4 ± 11.6	72.4 ± 9.2	0.331
**Heart rate (bpm) (mean± SD)**	80.6 ± 16.9	79.5 ± 16.5	0.742
**Echocardiographic findings**			
**Left atrium diameter (mm) (mean± SD)**	53.9 ± 6.9	38.9 ± 2.5	<0.0005
**LVEF (%) (mean± SD)**	39.5 ± 4.4	60.0 ± 4.9	<0.0005
**Cardiac diseases (n)**			
**Coronary heart disease**	29		
**Valvular diseases**	5		
**Diastolic dysfunction**	9		
**Dilatated Cardiomyopaty**	4		
**Total cholesterol (mMol/L) (mean± SD)**	136.5 ± 36.0	159.9 ± 40.7	0.003
**LDL cholesterol (mMol/L) (mean± SD)**	78.5 ± 32.9	86.6 ± 33.5	0.225
**Triglycerides (mmol/L) (mean± SD)**	82.7 ± 24.6	97.5 ± 50.1	0.069
**HBa1C (mean± SD)**	6.4 ± 1.1	6.0 ± 1.2	0.079
**Micro albuminuria (mean± SD)**	135.5± 208.2	60.2 ± 87.2	0.019
**RHI (mean± SD)**	1.5 ± 0.4	2.0 ± 1.0	0.002
**IL-18 pg/mL (mean± SD)**	477.2 ± 149.1	412.1 ± 186.7	0.059
**sIL2Ralfa pg/mL (mean± SD)**	964.9 ± 334.6	782.4 ± 419.6	0.020
**TNF-alfa pg/mL (mean± SD)**	8.6 ± 1.9	7.2 ± 3.7	0.021
**ACE inhibitors or ARB (n/%)**	31/65,9	30/56,6	0.339
**Beta blockade (n/%)**	29/61.7	21/39.6	0.028
**Calcium channel blockers (n/%)**	9/19.14	17/32	0.141
**Antiarrhythmic drugs (n/%)**	12/25.5	15/28.3	0.805
**Antiplatels drugs (n/%)**	25/53.1	12/22.6	0.002
**Anticoagulant drugs (n/%)**	27/57.4	26/49	0.401
**Diuretic (n/%)**	31/65	18/33.9	0.001
**Statin (n/%)**	18/38.3	16/30.2	0.393

**Figure 2:**
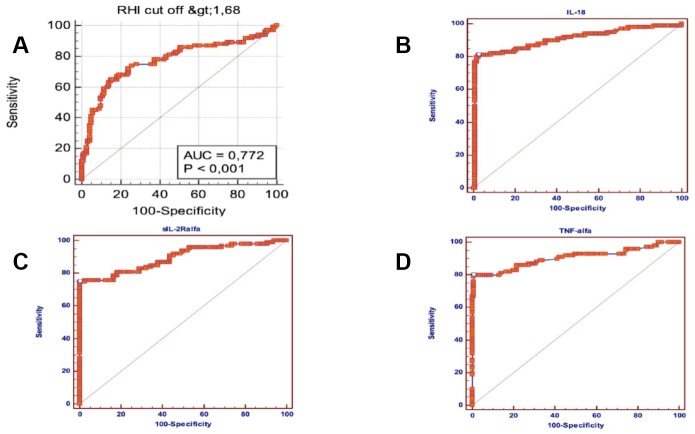
**Area under ROC *curve*, sensitivity and specificity of sIL-2Rα, IL-18, TNF-α and RHI in atrial fibrillation subjects.** (**A**) Area under ROC *curve*, sensitivity and specificity of reactive hyperaemia index (RHI) in atrial fibrillation subjects. (**B**) Area under ROC curve, sensitivity and specificity of IL-18 in atrial fibrillation subjects. (**C**) Area under ROC curve, sensitivity and specificity of sIL-2Rα in atrial fibrillation subjects. (**D**) Area under ROC curve, sensitivity and specificity of TNF-α in atrial fibrillation subjects.

A Multiple logistic regression model, including all the significant baseline variables (P<0.05) predictive of AF, showed that RHI (OR=5.94; 95% CI = 0.93- 20.78; p = 0.053), IL- 18 (OR = 1.01; 95% CI = 1-1.019; p = 0.042), TNF α (OR = 5.1; 95% CI = 1.35-19.18; p = 0.016), sIL-2Rα (OR= 1.02; 95% CI = 1.007-1.038; p = 0.005) and hypertension (OR = 6,8; 95% CI = 2.1-20.98; p = 0.003) were independently associated with the arrhythmia (Table 3). Moreover ROC curves identified the best cut-offs of RHI (AUC =0.772; p = 0.0001; sensitivity =65%, specificity = 85%, cut-off value ≤ 1.75), IL-18 (AUC=0.906; p= 0.0001; sensitivity = 81%, specificity = 97.52%, cut-off value> 269.9), TNF-α (AUC = 0.904, p = 0.0001; sensitivity = 80%, specificity = 99.17%, cut-off value> 5, 55), sIL-2Rα (AUC = 0.895; p = 0.0001; sensitivity = 74.75%, specificity = 100%, cut-off value> 511) for the presence of AF (Figure 2).

**Table 3:** Multivariable logistic regression analysis of variables predictive of atrial fibrillation.

**Variables**	OR*	**95% Confidence interval**	**p**
**IL-18**	1.01	1-1.019	0.042
**sIL2Ralfa**	1.02	1.007-1.038	0.005
**TNF-alfa**	5.1	1.35-19.18	0.016
**Hypertension**	6.8	2.1-20.98	0.003
**RHI**	5.94	0.93-20.78	0.053
**Micro albuminuria**	1.03	1-1.05	0.007
**Heart rate**	0.83	0.72-0.95	0.015
**Antiarrhythmic drugs**	5.36	1.67-27.07	0.03
**Diuretics**	7.9	1.92-33.07	0.021

*adjusted data for BMI, dyslipidemia, diabetes, ACE inhibitors or ARB, Beta blockade, SBP and DBP.

Multiple logistic regression analysis considering the relationship between inflammatory variables and SHD showed that sIL2Ralfa (OR= -1.84; 95% CI 0.99-1; p =0.276), TNF-α (OR= -1.75; 95% CI 0.92-1.03; p =0.286) and IL-18 (OR= -1.84; 95% CI 0.99-1; p =0.66) were not associated with the SHD presence (Table 4). At multiple logistic regression analysis of the relationship between inflammatory variables and vascular health markers such as RHI we found that sIL2Ralfa (OR=7.607; 95% CI=0.99-1; p <0.001), TNF-α (OR=7.106; 95% CI 0.39-0.91; p <0.01) and IL-18 (OR= 7.61; 95% CI 0.99-1; p =0.001) were associated with lower RHI values (RHI<2) (Table 5).

**Table 4:** Multivariable logistic regression of inflammatory variables predictive of SHD in atrial fibrillation patients.

**AF with SHD**	**Variables**	OR*	**95% Confidence interval**	**p**
	IL-18	-1.84	0.99-1	0.66
	sIL2Ralfa	-1.84	0.99-1	0.276
	TNF-alfa	-1.75	0.92-1.3	0.286

*adjusted data for BMI, dyslipidemia, diabetes, ACE inhibitors or ARB, Beta blockade, SBP and DBP.

**Table 5:** Multivariable logistic regression of inflammatory variables predictive of RHI<2 in atrial fibrillation patients.

**Variables**	OR*	**95% Confidence interval**	**p**
**IL-18**	7.61	0.99-1	0.001
**sIL2Ralfa**	7.607	0.992-0.998	0.001
**TNF-alfa**	7.106	0.39-0.91	0.019

*adjusted data for BMI, dyslipidemia, diabetes, ACE inhibitors or ARB, Beta blockade, SBP and DBP.

## DISCUSSION

In our study, we found that the "inflammatory cytokines milieu" (in our study defined as elevated IL-18, sIL-2Rα and TNF-α) of permanent AF subjects is significantly associated with arrhythmia but not with an underlying cardiovascular disease. To date, only a few studies have tried to address whether AF or the underlying SHD is the cause of the inflammatory response. For example, in the study conducted by Ellinor et al. [7] authors showed that patients with AF and hypertension had higher circulating CRP levels, compared with those of healthy controls (no AF group) and those of patients with LAF. Nevertheless, no differences were reported between patients with LAF and healthy controls regarding CRP levels, and therefore authors assumed that systemic inflammation is not associated with the AF itself, but rather with the underlying SHD. In another study, Pellegrino et al. [8] found that AF patients with underlying cardiovascular disease had higher CRP levels than LAF subjects. However, CRP levels in LAF patients were higher than those seen in healthy controls, suggesting that inflammation could be related to both conditions (AF and SHD). These studies investigated the inflammatory pathway of AF patients only by determining CRP plasma levels, but growing evidence suggests that the "inflammatory milieu" documented in AF subjects is more complex and involves various inflammatory markers, other than CRP [2–4].

Moreover, in consideration of our current state of knowledge, to date, the use of the historical term 'lone AF' is not recommended [9] and current guidelines [1] do not mention it. Unlike previous studies, we sought to assess more extensively the inflammatory process of AF patients with and without an underlying SHD.

Considering that our cohort consisted of patients with permanent AF, we sought to investigate the circulating levels of inflammatory markers related to this clinical subtype of AF.

Accumulating evidence supports the notion that tumor necrosis factor-alpha (TNF-α) is involved in the pathogenesis of chronic AF [18]. TNF-α plays paramount roles in atrial remodeling, including structural, electrical, contractile, and autonomic remodeling and higher TNF-α concentrations have been reported [19] in patients with persistent and permanent AF than in patients with paroxysmal AF supporting the contributes of this proinflammatory cytokine in the development of permanent forms.

Interleukin (IL)-18 is a pleiotropic proinflammatory cytokine with a central role in the inflammatory cascade. Besides, Interleukin-18 can induce TNF-α and IL-6 production by murine macrophages [20], and it can also enhance the expression of matrix metalloproteinases (MMPs) [21].

Higher levels of Interleukin-18 have been reported [22] in persistent AF patients than in paroxysmal ones supporting that interleukin-18 can mediate repair and remodeling of myocardium through increasing MMPs activity.

IL-2 is a proinflammatory cytokine that is primarily produced by activated CD4+ T cells, CD8+ T cells, and dendritic cells. There are scarce data on the relationship between IL-2 and AF [23]. The interleukin-2 (IL-2) receptor complex is an αβγ trimer, in which all three chains are in contact with the ligand. The α subunit of this complex, IL-2Rα is a 55-kilodalton transmembrane glycoprotein with only 13 amino acids of 351 located on the cytoplasmic side of the membrane. The function of the sIL-2Rα is unclear, but increased levels of the soluble IL-2Rα in biological fluids reportedly correlate with increased immune system activation suggesting that it is a reliable biomarker for disease activity in inflammatory disorders [24].

However, the circulating concentration of sIL-2Rα has not been reported in patients with AF, and thus, in this study, we sought to determine whether sIL-2Rα was associated with AF.

Our data suggest that systemic inflammation (in terms of IL-18, sIL-2Rα, and TNF-α) is not associated with the underlying SHD, but rather with the arrhythmia “per se.” This finding is furtherly confirmed by our logistic regression analysis showing no relationship between inflammatory markers such as selected cytokines serum levels and SHD.

We assume that this finding is attributable to the extremely peculiar nature of the arrhythmia that is able to induce many changes, consistent with a substantial cellular insult caused by excessively rapid activation. These include the oxidative injury of myofibrillar proteins in atrial myocytes, cellular myolysis, cardiomyocyte apoptosis and a local myocardial inflammation followed by a structural and electrophysiological remodeling which increase the persistence of AF itself [6, 25, 26]. To the best of our knowledge, this is a novel finding that further emphasizes the strong relationship between arrhythmia with the inflammatory process independently of underlying abnormality of heart structure.

We also reported that subjects with permanent AF had higher mean serum values of inflammatory cytokines in comparison to control subjects in sinus rhythm and without any history of AF. This finding suggests the presence of inflammatory activation in AF patients and is consistent with the growing evidence linking inflammation to AF [2]. It should be considered that our AF patients had a higher prevalence of some comorbidities such as hypertension, diabetes, and dyslipidemia. Furthermore, the percentage of patients taking antiarrhythmic drugs, anti-coagulants or diuretic is considerably higher in the AF cohort. The possible contribution of these conditions and pharmacological intervention in modulating the inflammatory process should be taken into account in interpreting these results. However, our findings on multinomial analysis concerning the significant association between the inflammatory markers and the arrhythmia corroborate the strong linking between the inflammatory process and AF. Moreover, ROC curve analysis had a good sensitivity and specificity of inflammatory cytokines to predict AF, and this further emphasizes the hypothesis that the inflammatory process is closely connected to AF.

In comparison to control subjects without arrhythmia, we also found that subjects with AF have an endothelial function impairment, and this result confirms what was previously reported in several studies [10, 11]. Associated co-morbidities such as hypertension, diabetes, and hypercholesterolemia were prevalent in our AF subjects. It is acknowledged that the presence of these co-morbid conditions may still be contributory to endothelial dysfunction and the influence of these conditions on our findings cannot be entirely discounted, although our multiple logistic regression analysis showed a significant association between the arrhythmia and RHI. Furthermore, on ROC curve analysis, there was also a had good sensitivity and specificity of RHI for AF presence.

Although our study could not prove a cause and effect relationship between AF and endothelial dysfunction, there are several pathogenic and plausible mechanisms for the connection. A possible pathogenetic explanation is that AF is characterized by rapid and uncoordinated atrial activity, causing ineffective atrial contraction with resultant turbulent flow both in the left atrium and systemically. Indeed, loss of shear stress, as occurs in conditions of turbulent flow, is associated with decreased endothelial nitric oxide synthase (eNOS) expression as compared with laminar or pulsatile flow conditions [27]. Another suggestive hypothesis is related to the higher degree of systemic inflammation observed in permanent AF subjects. According to this hypothesis, circulating inflammation mediators such as TNF-α and IL-18 that are strongly implicated with endothelial dysfunction and cardiovascular disease [28–30] might induce vascular damage and exacerbate the atherosclerotic burden leading to higher cardiovascular morbidity and mortality that is seen in AF subjects [1]. This finding is furtherly confirmed by our logistic regression analysis showing that inflammatory markers were associated with lower RHI values (RHI<2). Moreover, no study has examined endothelial function using reactive hyperemia peripheral arterial tonometry (RH-PAT) in a cohort of permanent AF subject; thus, our finding appears original.

Our study provides further evidence about the pivotal role of the inflammatory process in subjects with AF and suggest that the "inflammatory milieu" observed in patients with AF is the mirror of the arrhythmia 'per se', regardless of the presence of an underlying SHD.

Besides this, our findings suggest possible practical consequences in terms of emphasizing the use of drugs with pleiotropic and anti-inflammatory effects such as statins and renin-angiotensin- system (RAS)-inhibitors to modulate inflammatory pathways in AF subjects to prevent the arrhythmia and the complications related to it. The possible role of inflammation as a guide to conventional or anti-inflammatory therapy is further sustained by recent studies [31–34] that highlight the strong association of inflammation with cardiovascular events and its influence on the pathogenesis of the vascular disease. Definite conclusions cannot be drawn at this point and randomized controlled trials are needed to clarify these issues, but the idea that inflammation markers may be used as a guide to therapy is fascinating and reasonable.

### Limitations

This study is limited by the variability in medication used between AF subjects and controls. The cross-sectional analysis is a limitation since it does not allow conclusions on cause-effect relationships and the relatively small sample size may also limit conclusions. In our study subjects with AF showed higher levels of HbA1c, cholesterol and microalbuminuria compared with controls. To date, several studies suggest an association of inflammatory markers with urinary albumin excretion in the microalbuminuric range in type 2 diabetic and nondiabetic individuals and the association of worse glycemic and lipid control and greater systemic inflammation in the diabetic population. Moreover, inflammation plays a key role in all stages of the formation of vascular lesions included endothelial dysfunction. Therefore it is possible that these parameters are correlated with both inflammation and endothelial function.

## MATERIALS AND METHODS

Between March 2016 and October 2017, all consecutive patients with permanent AF admitted to our ward of Internal Medicine for a new episode of dysrhythmia or other conditions not related to arrhythmia, were enrolled in this study. The diagnosis of AF was made based on at least one 12-lead electrocardiogram and according to the current guidelines [1]. Patients with AF of >12 months in duration and ≥ 1 attempt at electrical cardioversion to restore normal sinus rhythm were considered to have permanent AF.

Hypertension was defined according to the 2013 ESH/ESC guidelines [13]. Type 2 diabetes mellitus was determined using a clinically based algorithm that considered age at onset, presenting weight and symptoms, family history, onset of insulin treatment, and history of ketoacidosis [14]. Hypercholesterolemia was defined as total serum cholesterol ≥200mg/dL, and hypertriglyceridemia as total serum triglyceride ≥150mg/dL on the basis of the National Cholesterol Education Program–Adult Treatment Panel III reports [15].

Among AF subjects, the presence of a structural heart disease (SHD) was defined by the following characteristics: valvular heart disease, hypertrophic cardiomyopathy, dilated cardiomyopathy, coronary artery disease, systolic dysfunction, diastolic dysfunction and key structural alterations such as left ventricular hypertrophy (LVH) and left atrial enlargement (LAE) these last two defined by the presence of a left atrial volume index (LAVI) >34 mL/m^2^ or a left ventricular mass index (LVMI) ≥115 g/m^2^ for males and ≥95 g/m^2^ for females according to the ESC guidelines for the diagnosis and treatment of acute and chronic heart failure [16]. Subjects free from the above-mentioned characteristics were considered without SHD.

As controls, among the consecutive subjects admitted to our ward between March 2016 and March 2018, we enrolled those who where matched to AF patients on the basis of age and gender, but in sinus rhythm and without any history of AF. Exclusion criteria were neoplasia, rheumatologic disorders (e.g., rheumatoid arthritis), other chronic inflammatory disease or a history of hospitalization, infection, or acute inflammatory illness within the past 6 weeks.

For AF subjects and controls we evaluated medical history, 12-lead ECG, and two-dimensional and pulsed Doppler echocardiography at admission.

### Clinical and laboratory assessment

Blood samples were collected from the antecubital vein after 10 minutes of rest in the supine position. In the AF cohort blood samples were withdrawn while the patients were in AF rhythm. We evaluated circulating plasma levels of soluble Interleukin 2 Receptor Alpha (sIL-2Rα), TNF-α and IL-18. sIL-2Rα and TNF-α were measured in plasma by ELISA (R&D Systems, Minneapolis, MN, USA); IL-18 were measured using a commercial sandwich ELISA (MBL, Medical and Biological Laboratories Co., Ltd, Nagoya, Japan). The minimum detectable concentrations for the diagnostic tests are: sIL-2Rα: 312 – 20,000pg/mL; IL-18: 12.5 pg/ml; TNF-α: 0.191 pg/mL. Intraassay and interassay coefficients of variation were: sIL-2Rα: 5,11-7,59%; IL-18: 10 -15 %; TNF-α 5.2-8.5%

### Rh-pat

The principle of RH-PAT has been described previously by researchers [17]. Briefly, a blood pressure cuff was placed on 1 upper arm, while the contralateral arm served as a control. PAT probes were placed on 1 finger of each hand. After a 5-min equilibration period, the cuff was inflated to 60 mm Hg above the systolic pressure or 200 mm Hg for 5 min and then deflated to induce reactive hyperemia. The RH-PAT data were digitally analyzed online (Endo-PAT2000 software version 3.0.4). The RH- PAT index reflects the extent of reactive hyperemia and was calculated as the ratio of the average amplitude of PAT signal over 1 min starting 1.5 min after cuff deflation (control arm, A; occluded arm, C) divided by the average amplitude of the PAT signal of a 2.5-min time period before cuff inflation (baseline) (control arm, B; occluded arm, D). Thus RH-PAT index (RHI) = (C/D)/(A/B) × baseline correction.

In this study, a standard approach was used to obtain the RHI measures. RHI values were recorded with patients in a supine and resting position. In the AF cohort, RHI measures were obtained in AF rhythm.

### Statistical analysis

Statistical analysis of quantitative and qualitative data, including descriptive statistics, was performed for all items. Continuous data are expressed as mean ±SD, unless otherwise specified. Baseline differences between groups were assessed by the chi-square test or Fisher exact test, as needed for categorical variables, and by the independent Student t test for continuous parameters if the data were normally distributed. Logistic regression analysis examined the correlation between patient characteristics (independent variables), and patient groups (dependent variable) in a simple model. All variables with a p-value lower than 0.5 at univariate analysis were ran in a multivariate linear regression model and the data were adjusted for BMI, dyslipidemia, diabetes, ACE inhibitors or ARB, Beta blockade, SBP and DBP. To assess the predictive rate of different cutoff values of inflammatory cytokines and RHI indexes with regard to patient groups, a receiver operating characteristic (ROC) curve with calculations of area under the curve and 95% CI was constructed, and sensitivity and specificity values were calculated. Data were analysed by IBM SPSS Software 22 version (IBM Corp., Armonk, NY, USA). All p-values were two-sided and p<0.05 was considered statistically significant.
